# Effects of Expressive Arts–Based Interventions on Adults With Intellectual Disabilities: A Stratified Randomized Controlled Trial

**DOI:** 10.3389/fpsyg.2020.01286

**Published:** 2020-06-11

**Authors:** Rainbow T. H. Ho, Caitlin K. P. Chan, Ted C. T. Fong, Pandora H. T. Lee, Derek S. Y. Lum, S. H. Suen

**Affiliations:** ^1^Department of Social Work and Social Administration, The University of Hong Kong, Pokfulam, Hong Kong; ^2^Centre on Behavioral Health, The University of Hong Kong, Pokfulam, Hong Kong; ^3^Madam Lo Lee Pui Ching Memorial Workshop, Yan Chai Hospital, Tsuen Wan, Hong Kong

**Keywords:** aberrant behavior, Chinese, emotional expression, expressive arts, intellectual disability, mood, stratified randomization

## Abstract

**Background:**

People with intellectual disabilities have difficulties expressing their views and can manifest psychological and behavioral symptoms. The present study aimed to examine the effects of expressive arts–based intervention (EABI) on the behavioral and emotional well-being of adults with intellectual disabilities.

**Methods:**

This study assigned 109 Chinese adults with intellectual disabilities into EABI (*N* = 55) or control groups (*N* = 54) using stratified randomization. Pre- and post-intervention quantitative assessments were conducted of aberrant behaviors, personal well-being, mood and color usage in drawings. Focus group interviews were conducted with the EABI participants and their caseworkers at the post-intervention stage. Repeated-measures analysis of covariance evaluated the EABI effects with age, gender and degree of intellectual disability as covariates, and latent profile analysis examined the patterns of color usage in drawings. Qualitative thematic analysis was performed on the interview data.

**Results:**

The interview findings suggest that the EABI group was more emotionally expressive and stable after the intervention. Compared to the control group, the EABI group tended to use more diverse colors and leave less empty space in their drawings. No significant overall improvements were found in the EABI group with respect to aberrant behaviors, mood or personal well-being. Among males, the EABI participants showed significantly more anger and less energetic moods than those in the control group. Among females, the EABI participants showed significantly lower levels of aberrant behavior than those in the control group.

**Conclusion:**

The results of this study suggest that expressive arts–based interventions have different effects on the emotional and behavioral well-being of male and female participants. Moreover, increased color usage may imply a more positive state of emotional well-being.

## Introduction

Intellectual disability (ID) is characterized by considerable deficits in cognitive, functional and adaptive life skills, with an IQ score of 70 or below (Schalock et al., 2010). People with ID generally tend to have difficulty expressing their thoughts and feelings verbally, which increases the likelihood that they will display their emotions through maladaptive behaviors (e.g., agitated, aggressive and self-injurious behaviors), which causes disruption to social relationships (Antonacci et al., 2008; Got and Cheng, 2008). Unfortunately, resources and support for this population in Hong Kong are limited, particularly for adults (Got and Cheng, 2008). This is because Chinese culture and society emphasize academic and vocational success (Stevenson and Lee, 1996) and tend to have low expectations and hopes for adults with ID, which undoubtedly makes them more vulnerable to psychosocial problems. Mcgillivray and Mccabe (2007) found that 39% of 151 sampled adults with ID displayed depressive symptoms with contributing factors such as negative thoughts, social exclusion and disruptive life events. As body gestures, sounds and pictures are effective non-verbal means of human communication (Phutela, 2015), arts-based interventions may be a useful and viable way to help this underserved group.

According to expression theories of art, all forms of art involve “bringing feelings to the surface, bringing them outward where they can be perceived by artists and audiences” (Carroll, 2002). Through externalizing the inner emotional state, the arts make discoveries about the emotions and help transmit feelings and/or messages to others (Carroll, 2002). Evidence shows that arts-based interventions such as music therapy (Duffy and Fuller, 2000; Poquérusse et al., 2018), mandala making (Schrade et al., 2011) and art facilitation (Got and Cheng, 2008) can enhance the well-being of people with ID in terms of reduced stress and enhanced language comprehension.

Compared to interventions based on a single art modality, expressive arts–based intervention (EABI) emphasizes the integrative use of various arts modalities (e.g., visual arts, dance/movement and music). It not only aims to cater to clients’ individual needs and enhance their curiosity and playfulness, but also rests on the intermodal theory which suggests that emotional expression may be accentuated by shifting from one art modality to another (Knill et al., 1995). Such process helps deepen the exploration and expression of the emotions, and provides opportunity for the participants to change their perspectives. To the best of our knowledge, although EABI has shown beneficial effects on the psychosocial well-being of various groups, such as students (Aaron et al., 2011), children in need (Coholic et al., 2012), family caregivers (Walsh et al., 2004), teachers (Ho et al., 2012) and domestic violence survivors (Lai, 2011), no empirical research has been conducted on adults with ID in any context. Given the increasing use of arts intervention in healthcare settings, there is an urgent need for evidence-based research to assess its effectiveness.

The objective of the present study was to provide a first evaluation of the effectiveness of EABI on the behavioral, emotional and personal well-being of adults with ID using a mixed-methods randomized controlled trial design. The experimental design with a wait list control group yields rigorous empirical evidence regarding the effectiveness of the intervention program, while utilizing both quantitative and qualitative data broadens our understanding of the effectiveness of the intervention. It was expected that EABI would facilitate emotional expression among these individuals. Quantitative data generated from the study inform us about the program’s effectiveness, while the qualitative data enrich our understanding of the underlying mechanisms and facilitate the building of an evidence-based service model for further service development.

## Methods

### Participants and Procedures

The present study adopted a stratified randomized controlled design with pre- and post-intervention measurements. The participants were service users of six residential healthcare or community-based rehabilitation support service centers of a local NGO in Hong Kong. Those eligible to participate in the intervention program were individuals with mild to moderate levels of ID (as assessed by a mental health professional) who were receiving either residential healthcare or community-based support services at the time of enrollment. A total of 120 eligible service users were selected and referred by the NGO’s caseworkers using convenience sampling from August 2017 to January 2018. Written informed consent was distributed to their guardians. Eleven of them declined to join the study, resulting in 109 eligible participants being recruited into the study. The recruited participants were first stratified in accordance with their gender and degree of ID and then assigned to either the EABI group (*n* = 55) or the wait list control group (*n* = 54) using simple randomization conducted by a research assistant.

Because there was potential treatment heterogeneity across these two factors (male/female and mild/moderate impairments), stratified randomization may help to avoid an imbalance between the treatment groups in terms of factors that could affect treatment responsiveness. In addition, stratification has shown improved power in clinical trials with a small sample size (Kernan et al., 1999). As shown in the CONSORT flowchart in Supplementary Figure S1, there were no dropouts in either group.

The EABI group was invited to participate in a 10-week intervention during the September 2017 to April 2018 period. Quantitative assessments were conducted before and after the intervention, while qualitative evaluation was done at the post-intervention stage. Upon completion of all evaluation exercises, the wait list control group received a comparable intervention. The study was independently reviewed and approved by the human research ethics committee of the local university and conformed to the recognized standards of the Declaration of Helsinki. The mean age of the sample was 39.9 years old (SD = 12.4). Half of the participants were female (54%) and had a mild degree of ID (51%). As shown in Table 1, the two groups did not differ significantly (*p* = 0.39–0.92) in these characteristics.

**TABLE 1:** Demographic and baseline characteristics of the participants by group.

	Intervention	Control		
	*N* (%)	*N* (%)	χ^2^	*p*
Gender – *Female*	28 (51)	31 (57)	0.46	0.50
*IQ* – *Mild impairment*	26 (47)	30 (56)	0.75	0.39

	***Mean (SD)***	***Mean (SD)***	***t***	***p***

Age (years)	39.8 (12.1)	40.0 (12.7)	−0.10	0.92
**Aberrant behavior**				
Lethargy	4.4 (6.9)	2.5 (4.0)	1.75	0.08
Irritability	5.7 (7.8)	4.8 (9.1)	0.53	0.60
Hyperactivity	2.5 (3.9)	2.8 (5.5)	−0.30	0.77
Self-injurious behavior	1.5 (2.9)	1.0 (2.9)	0.95	0.34
Inappropriate speech	1.2 (1.8)	1.4 (2.2)	−0.39	0.70
**VAMS**				
Afraid	28.5 (40.3)	28.7 (36.9)	−0.03	0.98
Confused	34.0 (41.9)	29.7 (37.9)	0.56	0.58
Sad	29.6 (37.5)	28.4 (36.5)	0.17	0.87
Angry	21.0 (35.8)	30.5 (40.1)	−1.30	0.20
Energetic	84.8 (28.7)	75.4 (36.7)	1.49	0.14
Tired	38.5 (40.2)	40.3 (40.6)	−0.23	0.82
Happy	79.2 (34.1)	79.7 (32.5)	−0.07	0.95
Tense	29.1 (39.7)	26.0 (36.8)	0.41	0.68
Personal well-being	7.9 (1.8)	7.7 (2.4)	0.44	0.66

**N* = 109; χ^2^, chi-square; *t*, independent-sample *t*-test.*

### Intervention

The EABI group received 10 weekly 90-min sessions, with a total of 15 contact hours. Each intervention group contained 6–8 participants and was facilitated by a registered expressive arts therapist or expressive arts therapy trainee. The EABI program was developed in accordance with the literature on arts-based interventions (Maujean et al., 2014) and the psychosocial needs of people with ID (Malley et al., 2002; Got and Cheng, 2008), as well as the clinical experiences of arts therapists and other healthcare professionals in the service units. Given the limited capacity for verbal expression of the participants, the program aimed to facilitate their emotional awareness and expression through various art forms such as visual arts, dance/movement and music. Linkages between emotions and elements of different art forms (e.g., color, shape, sound, and body movement) were explored in different sessions.

Each session began with greeting the participants and introducing the session theme. Simple body movements or music games were used as warm-up exercises to build rapport and encourage creativity among the participants. Next, they were guided through a process of improvisation of rhythm or dance/movement to raise their awareness of their emotions and stimulate expression. After that, art-making processes allowed the participants to create and/or revise artworks or music pieces that represented their thoughts and feelings. They were then encouraged to verbally share their art-making experiences. Through articulating the relationships between their art, the creative process and their emotions, the participants were able to understand and regulate their emotions better. Finally, the session ended with a closing ritual to consolidate the experience and build group cohesion. The theme and contents of each session are summarized in Supplementary Table S1.

The structure of each session was kept similar, while the content was adjusted to suit the immediate needs and dynamics of the group. The control group participated in routine healthcare and rehabilitation services. Neither the participants nor the therapists were blinded to the study condition assignment.

### Assessments

The study adopted a mixed-methods, repeated-measure study design with a wait list control group, utilizing both quantitative and qualitative data. The quantitative component used validated assessments, which were completed either by the participants or their mental health caseworkers via a paper-and-pencil format examining domains of the behavioral, emotional and personal well-being of the participants at two time points (baseline and post-intervention). The Aberrant Behavior Checklist (Rojahn et al., 2003) is a behavior-rating scale that was used to rate the participants with ID on a 4-point scale (0 = “Not at all,” 3 = “Severe”). This 58-item scale measures aberrant behaviors in five domains, namely, (1) lethargy (Sample items: “Inactive” and “Fixed expression, lacks response”), (2) irritability (Sample items: “Screams inappropriately” and “Demands must be met”), (3) hyperactivity (Sample items: “Impulsive” and “Disobedient”), (4) stereotypic and self-injurious behavior (Sample items: “Recurring body movements” and “Rolls head repetitively”) and (5) inappropriate speech (Sample items: “Repetitive speech” and “Talks to self loudly”). It was completed by the participants’ mental health caseworkers. All of the five subscales showed good to excellent reliability (α = 0.77–0.95) in the present study.

The Visual Analog Mood Scale (Stern et al., 1997) is a validated tool for assessing eight mood states (afraid, confused, sad, angry, energetic, tired, happy, and tense) in adults with ID. The test includes a neutral face at one end of a 10 cm vertical line and a specific “mood” face at the other end. Participants indicated the point along the line that corresponded to their current mood state. The score for each mood state ranges from 0 to 100.

The Personal Well-being Index (Sibley, 2009) is a self-reported, 8-item scale that assesses seven domains of quality of life, including standard of living, health, life achievement, personal relationships, personal safety, community-connectedness, future security and life satisfaction as a whole. The total score for this scale had a theoretical range from 0 to 10 and showed good reliability (α = 0.86) in the present study.

The Face Stimulus Assessment-Revised (Mattson, 2012) is a computerized arts-based assessment which has been proven to be a viable means of extracting psychological information about emotional expressions (Mattson and Betts, 2016) and distinguishing artwork by individuals with mental illness from those without through a face drawing task (Mattson, 2012). It was specifically designed for individuals with communication difficulties, including intellectual disability (Mattson and Betts, 2016). The assessment contains an outline of a half-drawn human face on a single sheet of A4 paper to allow the participants to project more information freely onto the image. A series of visual items also surround the face to encourage copying. The participants were given 30 min to color, draw or mark within the sheet using a Zebra^®^ Hi-McKee 12-colored permanent marker set. The artworks were digitally scanned, and the pattern of color usage in the pre- and post-intervention drawings was analyzed. We extracted the likelihood and intensity of using 12 colors: pink, red, orange, yellow, light green, green, light blue, blue, purple, light brown, brown, and black. Supplementary Table S2 shows the proportions of all participants using the 12 colors at the pre- and post-intervention measurements. The three most frequently used colors were pink, black and blue. Because none of the 109 participants used the light brown color in either measurement, this color was not considered in further analysis.

### Qualitative Evaluation

Two semi-structured focus group interviews were conducted separately with the participants from two sites (*n* = 10) who took part in the EABI program and one with their caseworkers (*n* = 9) at the post-intervention stage. Written informed consent for participating in the interviews was obtained from the participants’ guardians and caseworkers. An interview guide was developed (see Supplementary Appendix I) to gather a multi-faceted perspective on the process of change observed in the participants during their experience of the intervention. Each interview lasted for 60–90 min and was recorded on audiotape for transcription and later analysis. Thematic analysis was conducted using the computer software ATLAS.ti. To investigate the process of change observed in the participants during their experience in the study, the contents of the interviews were transcribed verbatim and coded line-by-line. Each transcript was analyzed in sentences or groups of sentences conveying single ideas. A code, using the interviewees’ wording, was first given to each idea. Similar ideas were grouped together. Through constant comparisons across ideas, some codes were renamed based on interviewees’ descriptions and elaborations. Codes with similar characteristics or natures were clustered into themes. To lower researcher bias, the analysis of the interview contents was conducted by two researchers (CKP and DSY) who coded the transcripts independently. Meetings were held between the two researchers to discuss and resolve discrepancies in assigning or grouping codes and themes. If disagreements could not be resolved, consultation with the project supervisor (RTH) was sought.

### Data Analysis

Independent *t*-tests and chi-square tests were used to compare the demographics and baseline characteristics of the groups. The outcome variables of the present study were the five subscales of aberrant behaviors (lethargy, irritability, hyperactivity, stereotypic and self-injurious behavior, and inappropriate speech), the eight mood states (afraid, confused, sad, angry, energetic, tired, happy, and tense) and the total score for personal well-being. To evaluate the intervention effects, a repeated-measures analysis of covariance was conducted using the group (EABI vs. control) as a between-subject factor and time (baseline vs. post-intervention) as a within-subject factor in SPSS 23. Age, gender and degree of ID were included as model covariates to control their potential confounding effects on the treatment. The clustering effect of participants within groups was taken into account in the analysis.

For the art-based assessment, artwork analysis was conducted using Image Java 1.49 (ImageJ). All of the drawings were converted into digital images by color scanning with a 600 dpi resolution and then imported into ImageJ. Median filtering was used to remove noise from the images that could alter the results of later analysis. The color thresholding technique was then applied to extract regions of each constituent color from the images. By specifying the range of HSB (hue, saturation and brightness) values, pixels of a certain color were extracted, and pixels of other colors were rejected. After segmenting the images based on the color information, the intensity (i.e., area) of each color used by the participants was calculated. Latent profile analysis was then performed to analyze the pattern of color usage and intensity for both the pre- and post-intervention drawings in the overall sample. Because of the smaller group size, we did not conduct the latent profile analysis separately in the two groups. We examined the potential sample heterogeneity of the color pattern and tested its association with the intervention group. Substantive checking of latent classes was conducted with respect to the model covariates, namely, age, gender, and degree of intellectual impairment.

To evaluate the potential heterogeneity of treatment effects in different subgroups, a secondary analysis was carried out to examine the subgroup-specific intervention effects across gender and degree of ID. The overall level of statistical significance was set at *p* = 0.05. The effect size of the intervention was denoted by the partial eta-squared (η^2^) with values of 0.01, 0.06, and 0.14 representing small, moderate, and large magnitude, respectively. Missing data was minimal (<2%) in the present study. The study dataset can be obtained from the corresponding author on request by email.

## Results

### Baseline Characteristics

Table 2 displays the descriptive statistics of the psychosocial variables (aberrant behavior, mood, and personal well-being) for the treatment groups before and after intervention. None of the demographic and outcome variables differed significantly (*p* = 0.08–0.98) across the EABI and control groups at baseline.

**TABLE 2:** Outcome variables of the participants by group before and after the intervention.

	**Intervention (*N* = 55)**		**Control (*N* = 54)**	
	***Pre***	***Post***	***Pre***	***Post***

	***Mean (SD)***	***Mean (SD)***	***Mean (SD)***	***Mean (SD)***
**Aberrant behavior**				
Lethargy	4.4 (6.9)	2.9 (5.2)	2.5 (4.0)	2.7 (4.5)
Irritability	5.7 (7.8)	3.4 (5.6)	4.8 (9.1)	3.7 (6.3)
Hyperactivity	2.5 (3.9)	1.8 (2.7)	2.8 (5.5)	2.3 (5.0)
Self-injurious behavior	1.5 (2.9)	0.8 (1.6)	1.0 (2.9)	0.9 (2.3)
Inappropriate speech	1.2 (1.8)	0.9 (1.4)	1.4 (2.2)	0.8 (1.5)
**VAMS**				
Afraid	28.5 (40.3)	29.2 (38.7)	28.7 (36.9)	25.9 (37.5)
Confused	34.0 (41.9)	17.2 (31.7)	29.7 (37.9)	14.3 (29.9)
Sad	29.6 (37.5)	17.8 (33.2)	28.4 (36.5)	21.2 (33.9)
Angry	21.0 (35.8)	26.7 (39.0)	30.5 (40.1)	23.1 (37.7)
Energetic	84.8 (28.7)	86.2 (26.0)	75.4 (36.7)	87.2 (24.8)
Tired	38.5 (40.2)	44.0 (41.6)	40.3 (40.6)	35.4 (41.4)
Happy	79.2 (34.1)	87.1 (24.1)	79.7 (32.5)	84.8 (24.6)
Tense	29.1 (39.7)	34.7 (41.0)	26.0 (36.8)	31.4 (42.0)
Personal well-being	7.9 (1.8)	8.4 (1.5)	7.7 (2.4)	8.3 (1.8)

*SD, standard deviation; χ^2^, chi-square; *t*, independent-sample *t*-test.*

### Overall Intervention Effects

Overall, no intervention effects (*F*_1_,_99_ = 0.02–0.34, *p* = 0.56 –0.89, η^2^ < 0.01) were found for EABI on personal well-being, hyperactivity or inappropriate speech. There was a small but statistically non-significant intervention effect (*F*_1_,_96_ = 2.15 –2.59, *p* = 0.11–0.15, η^2^ = 0.02–0.03) on lethargy, irritability, and stereotypic and self-injurious behaviors. Similarly, the EABI did not show any effects on most of the eight mood states (*F*_1_,_102_ = 0.01–1.74, *p* = 0.19–0.93, η^2^ < 0.02), except for feeling angry (*F*_1_,_102_ = 3.70, *p* = 0.06, η^2^ = 0.04).

### Subgroup Intervention Effects

**FIGURE 1:**
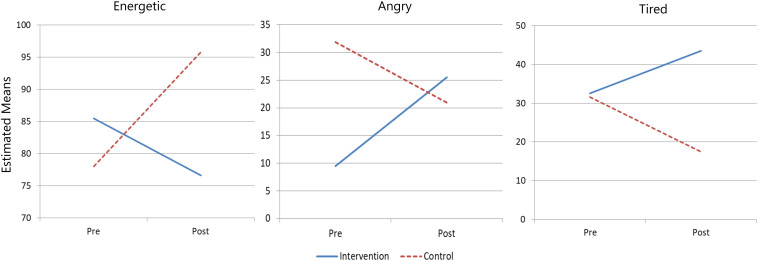
Group trajectories of feeling angry, energetic, and tired moods of male participants across time.

Subgroup analysis did not reveal any differential treatment effects for EABI across the degree of ID. Subgroup analysis across gender showed interesting findings in the male and female subsamples. Among males (*n* = 47), there were moderate to large effects on feeling angry (*F*_1_,_44_ = 9.94, *p* < 0.01, η^2^ = 0.18), energetic (*F*_1_,_44_ = 5.43, *p* = 0.02, η^2^ = 0.11) and tired (*F*_1_,_44_ = 4.13, *p* < 0.05, η^2^ = 0.08). Figure 1 shows the changes in these emotions for male participants. The EABI group reported more feelings of anger and tiredness but less energy after the intervention compared to the control group.

**FIGURE 2:**
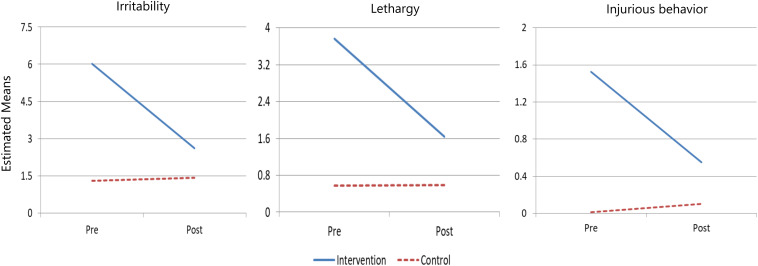
Group trajectories of lethargy, irritability, and stereotypic and self-injurious behaviors of female participants across time.

Among females (*n* = 57), the EABI showed significant and moderate effects on three aberrant behaviors: lethargy (*F*_1_,_48_ = 4.06, *p* < 0.05, η^2^ = 0.08), irritability (*F*_1_,_48_ = 6.54, *p* = 0.01, η^2^ = 0.12), and stereotypic and self-injurious behavior (*F*_1_,_48_ = 5.88, *p* = 0.02, η^2^ = 0.11). Figure 2 shows the changes in these aberrant behaviors for female participants. Females in the EABI group exhibited considerable decreases in these aberrant behaviors after the intervention compared to their counterparts in the control group.

### Art-Based Assessment

In the pre-measurement drawings, the majority of the participants (86%) showed consistently low probabilities of using any of the colors, apart from a minor subgroup (14%). These two subgroups did not, however, differ significantly in terms of the intervention group and other demographic characteristics (*p* = 0.39–0.96). In the post-measurement drawings, more than half of the participants (60%) belonged to the “pale subgroup,” and the remaining participants (40%) belonged to the “colorful subgroup.” Supplementary Figures S2, S3 show the subgroup profiles for the probability and intensity of using colors in the post-measurement drawings, respectively.

Participants in both subgroups showed similar likelihoods of using the orange, yellow, green and brown colors. The colorful subgroup was more likely to use the pink, blue and purple colors but less likely to use the black color than the pale subgroup. Moreover, the colorful subgroup (mean = 11.5 – 13.7) showed higher levels of intensity in using colors such as pink, red, green, and purple than the pale subgroup (mean = 10.1 – 12.3). Compared to the control group, participants in the EABI group were more likely (odds ratio = 2.42, 95% CI = 0.93 – 6.34) to belong to the colorful subgroup than to the pale subgroup, although this association did not reach statistical significance [χ^2^(1) = 3.49, *p* = 0.06].

### Qualitative Findings

The sharing of the personal experiences of the participants in the EABI group and their caseworkers’ daily observations revealed changes in the participants with respect to four aspects: (1) emotion wellness, (2) expressivity, (3) self-regulation, and (4) interpersonal relationships. In general, the intervention program helped promote behavioral and psychological well-being among the participants. They became more expressive in both verbal and non-verbal communications. A summary of the interview findings is presented in Supplementary Table S3.

## Discussion

The present randomized controlled trial is the first study to evaluate the effectiveness of EABI on the behavioral and psychological well-being and functioning of people with ID. The interview findings suggest that the participants in the EABI group were happier, calmer, more emotionally stable and less irritated after the intervention. They also became more capable of expressing their feelings and regulating their emotions and behaviors. Drawings by the EABI group at the post-intervention stage tended to be more colorful (in terms of color variety and colored area) compared to those by the control group. Despite the absence of overall intervention effects, subgroup analysis showed different intervention effects across gender. Compared to their control counterparts, male participants in the EABI group experienced a change in mood states (i.e., more anger, more tiredness and less energy) on treatment completion, while female participants in the EABI group exhibited fewer behavioral problems in terms of lethargy, irritability or stereotypic and self-injurious behavior. The results of the subgroup analysis, art-based assessments and focus group interviews triangulated with one another and suggested that EABI has potential benefits in terms of improving behavioral and emotional well-being among people with ID.

### Colors and Emotions

The relationship between color and emotions has long been a subject of interest in research (Jue and Kwon, 2013). Color usage, such as the number of colors used and the ratio of colored area versus empty space in a drawing, has been considered a possible visual indicator of emotional wellness. Limited use of colors and increased empty space have been found in drawings by people with histories of abuse, depression and chronic schizophrenia, compared to those created by members of a healthy control group (Gulbro-Leavitt and Schimmel, 1991; Cohen et al., 1994; Malchiodi, 1997; Gantt, 2001; Swan-Foster et al., 2003; Mattson, 2012; Jue and Kwon, 2013). The inclusion of art-based assessment in the present study could reduce potential bias caused by the limited verbal capacity of the participants throughout the data collection. Participants in the EABI group tended to apply and fill in more colors in their post-intervention drawings, and such a pattern of color usage was similar to that of the healthy control groups reported in previous studies. It is plausible that the EABI helped to enhance the emotional wellness of the participants. However, given the limited research on the usefulness of EABI for people with ID, more research is still needed to elucidate the relationship between color usage and emotional expression in people with ID.

### Gender Differences in Emotion Regulation

The findings of the subgroup analysis suggest different effects of the EABI on males and females with ID. They indicate that the EABI had a negative effect on the male participants and was only beneficial for female participants because the male participants in the EABI group reported higher levels of psychological distress at the post-intervention stage. However, such a difference may imply different stages of progression in emotion regulation between males and females. Because the EABI encouraged participants to express themselves in a non-judgmental manner, it increased the participants’ willingness to express different emotions. When emotions were expressed in various art forms, their exploration became easier and more concrete, which helped enhance the participants’ awareness of their emotions. The increased level of psychological distress reported by the male participants at the post-intervention stage may reflect higher levels of awareness of and willingness to express their emotions. According to emotion theories, emotional awareness plays an important role in effective emotion regulation (Boden and Thompson, 2015) because attending to and understanding one’s emotions can inform the selection and application of appropriate emotion regulation strategies (Barrett and Gross, 2001).

When comparing the outcomes across gender subgroups, the female participants seemed to have overcome the challenge of expressing and regulating their emotions, resulting in fewer behavioral problems. In contrast, the male participants could still be at the stage of attending to, understanding and expressing their emotions. This interesting finding was consistent with the well-established perspective on gender differences in emotional expression that suggests that, in general, men are less emotionally expressive than women (Nolen-Hoeksema, 2012). Socially acceptable displays of gender-specific stereotypical emotions are culturally instilled in males and females from early childhood (Parkins, 2012). Having an intellectual disability could pose additional difficulties for males in properly expressing their emotions. Due to the differences in emotional expressivity across gender, a higher intervention dosage may be considered for male participants in future studies. Further studies should also attempt to explore the mechanisms that underline the differential intervention effects of EABI across gender.

### Study Limitations

Several limitations of the present study should be noted. First, despite the stratified randomization design, the participants were recruited using convenience sampling, and there could be potential selection and sampling biases. Caution is warranted in attempting to generalize the present findings to other contexts until replications in future large-scale randomized control trials have been completed. Second, all of the participants were recruited from residential or community healthcare centers of the same local NGO. They could be under the common influence of organizational characteristics such as care policies, care culture or staff practices. These unmeasured organizational factors could potentially confound the current findings. Third, the present study only assessed the immediate treatment effects of the EABI before and after the intervention. Future longitudinal studies with a longer follow-up period are needed to evaluate the maintenance effect of EABI and its long-term effectiveness.

### Conclusion

This study contributed to the development of a feasible EABI program for people with ID that may raise the participants’ awareness of their own emotions, enhance their capacity to express their emotions and improve their behavioral well-being. The EABI group tended to use more colors in their post-intervention drawings. Such a pattern of color usage was similar to that in drawings created by healthy individuals in previous studies. Combined with the interview findings, the EABI may help improve the emotional wellness of people with ID. The different intervention effects across gender imply a gender difference in the progress of emotion regulation. The female participants seemed to have overcome the challenge of regulating their emotions, while the male participants were still at the stage of noticing and expressing their emotions. In this regard, a higher intervention dosage should be considered for male participants in future studies.

## Data Availability Statement

The raw data generated from this study will be made available by the authors, without undue reservation, to any qualified researcher.

## Ethics Statement

The studies involving human participants were reviewed and approved by Human Research Ethics Committee, The University of Hong Kong. The guardians of people with ID and caseworkers provided their written informed consent to participate in this study.

## Author Contributions

RH: conceptualization, validation, resources, writing – review and editing, supervision, and funding acquisition. CC: conceptualization, methodology, investigation, and writing – original draft. TF: software, formal analysis, data curation, writing – original draft, and visualization. PL: conceptualization, project administration, and writing – original draft. DL: investigation, writing – review and editing, and project administration. SS: resources, writing – review and editing, project administration, and funding acquisition. All co-authors participated in the writing of the manuscript.

## Conflict of Interest

The authors declare that the research was conducted in the absence of any commercial or financial relationships that could be construed as a potential conflict of interest.

## Supplementary Material

FIGURE S1 CONSORT flowchart of the study on EABI.

FIGURE S2 Subgroup profiles in the probability of using the 11 colors in post-measurement drawings.

FIGURE S3 Subgroup profiles in the intensity of using the 11 colors in log scale in post-measurement drawings.

TABLE S1 Themes and contents of the intervention program.

TABLE S2 Proportion and intensity of the respondents in using the twelve colors in the drawing before and after the intervention.

TABLE S3 Summary of the improvements found in the study participants.

## Abbreviations

EABI: expressive arts-based intervention
NGO: non-governmental organization.
