# Supplementary figures and images for: Effects of Expressive Arts–Based Interventions on Adults With Intellectual Disabilities: A Stratified Randomized Controlled Trial
Source: Front Psychol. 2020 Jun 11;11:1286. doi: 10.3389/fpsyg.2020.01286 (PMC7300289; doi:10.3389/fpsyg.2020.01286)

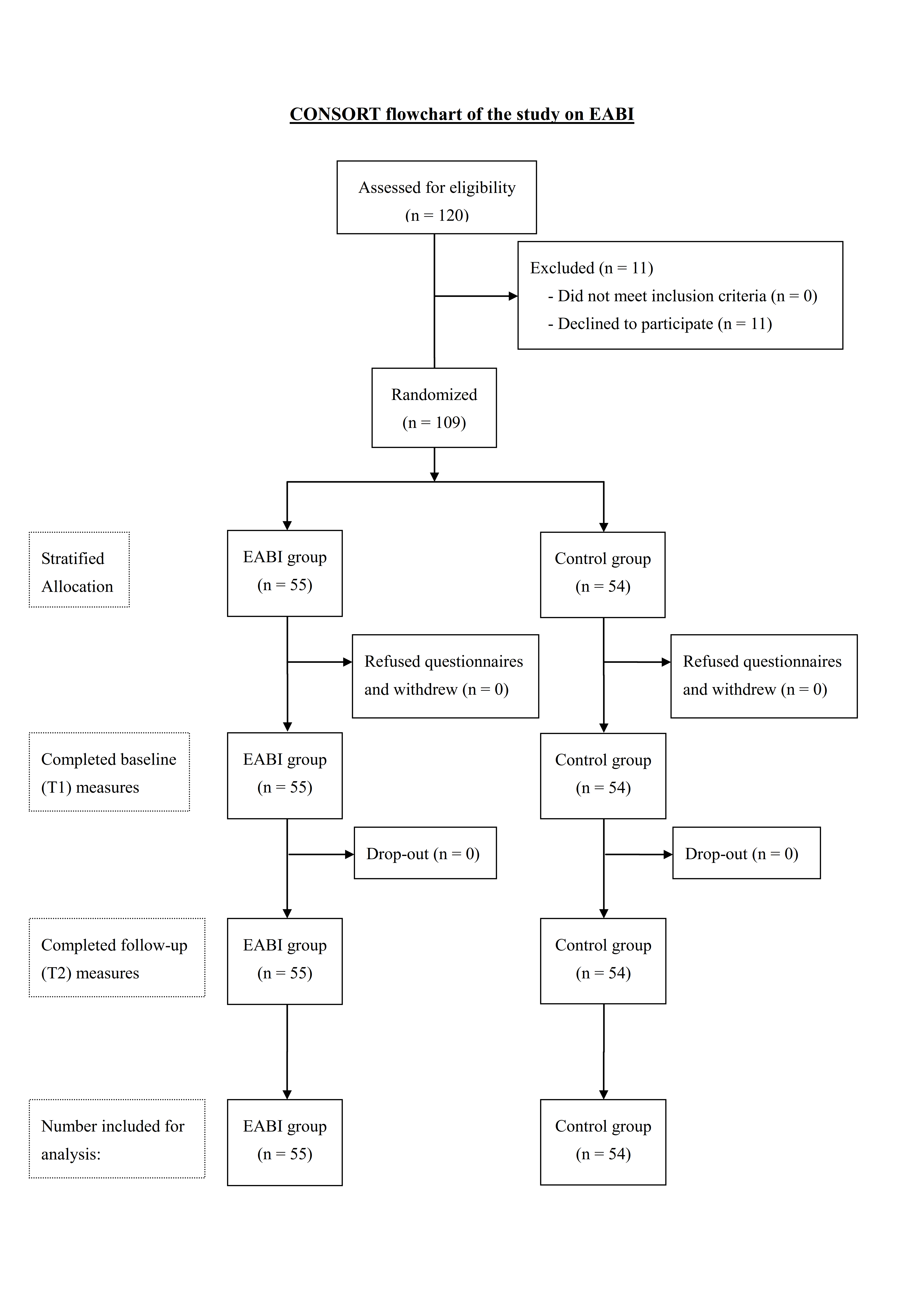

Supplement: FIGURE S1 — CONSORT flowchart of the study on EABI. [file Image_1.tif]

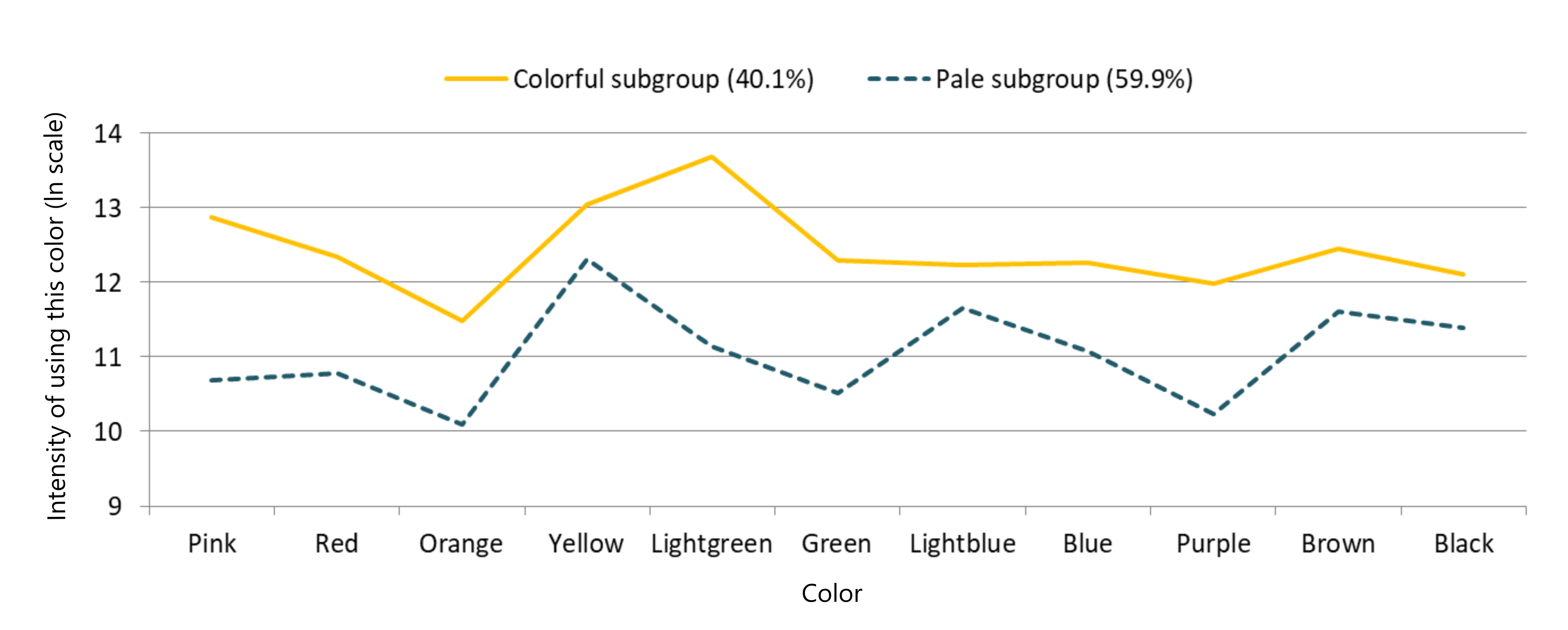

Supplement: FIGURE S3 — Subgroup profiles in the intensity of using the 11 colors in log scale in post-measurement drawings. [file Image_3.TIFF]
