# Supplementary material for: Effects of Expressive Arts–Based Interventions on Adults With Intellectual Disabilities: A Stratified Randomized Controlled Trial
Source: Front Psychol. 2020 Jun 11;11:1286. doi: 10.3389/fpsyg.2020.01286 (PMC7300289; doi:10.3389/fpsyg.2020.01286)
Supplement: TABLE S1 — Themes and contents of the intervention program. [file Table_1.docx]

**Appendix I**

Semi-structured interview guide

Note: The themes and interview questions will not be limited to the following and will be modified according to the participant’s responses and emerging themes.

1. In general, how would you (your clients) comment on this EABI program?
2. How do you (your clients) feel about the art activities done in the program? Any impressive memories?
3. How would you describe your (your clients’) relationship with family, colleagues and/or friends before and after joining the program?
4. How would you (your clients) regulate your (their) emotions? Can you share the new methods you (your clients) learnt from the program, if any?
5. What did you (your clients) gain from the program?
