# Supplementary material for: Effects of Expressive Arts–Based Interventions on Adults With Intellectual Disabilities: A Stratified Randomized Controlled Trial
Source: Front Psychol. 2020 Jun 11;11:1286. doi: 10.3389/fpsyg.2020.01286 (PMC7300289; doi:10.3389/fpsyg.2020.01286)
Supplement: TABLE S2 — Proportion and intensity of the respondents in using the twelve colors in the drawing before and after the intervention. [file Table_2.docx]

| **Supplementary Table 1.** Themes and contents of the intervention program | | |
| --- | --- | --- |
| **Session** | **Theme** | **Contents** |
| 1 | Self-introduction | To introduce group norms and ways to express using arts |
| 2 | Emotion awareness | To be aware of one’s own emotions through movement and arts creation |
| 3 | Emotion expression | To express one’s own feelings and emotions through sounds and voices |
| 4 | Emotion soothing | To soothe one’s own emotion through art making |
| 5 | Emotion regulation | To create one’s own emotion garden |
| 6 | Communication with others | To communicate with other through creating artwork with others |
| 7 | Cooperation with others | To cooperate with other through creating music with others |
| 8 | Personal expression | To express oneself through music and visual art |
| 9 | Connection through arts | To connect each group member through arts |
| 10 | Review | To review the group process and one’s own growth, as well as to give blessings to each other |

| **Supplementary Table 2.** Proportion and intensity of the respondents in using the twelve colors in the drawing before and after the intervention | | | | |
| --- | --- | --- | --- | --- |
|  | Before intervention | | After intervention | |
| Color | % | *M* (*SD*) | % | *M* (*SD*) |
| Pink | 45.3 | 12.3 (1.5) | 40.7 | 11.8 (1.4) |
| Red | 18.9 | 11.8 (1.3) | 22.2 | 11.2 (1.0) |
| Orange | 15.1 | 11.9 (1.3) | 10.2 | 10.9 (1.2) |
| Yellow | 15.1 | 11.9 (1.3) | 21.3 | 12.6 (1.4) |
| Light green | 21.7 | 12.4 (1.2) | 16.7 | 12.0 (1.3) |
| Green | 8.5 | 11.8 (1.0) | 7.4 | 11.2 (1.0) |
| Light blue | 11.3 | 11.5 (1.3) | 10.2 | 12.2 (0.6) |
| Blue | 37.7 | 11.9 (1.2) | 31.5 | 11.8 (1.2) |
| Purple | 8.5 | 12.1 (1.3) | 19.4 | 11.3 (1.1) |
| Light brown | 0 | 0 | 0 | 0 |
| Brown | 14.2 | 11.9 (1.5) | 16.7 | 11.9 (1.5) |
| Black | 33.0 | 11.2 (1.6) | 35.2 | 11.6 (1.2) |

| **Supplementary Table 3.** Summary of the improvements found in the study participants | | |
| --- | --- | --- |
| **Themes** | **Codes** | **Quotations** |
| Emotion wellness  (N=7) | Calmer  (n=3) | When they were in the expressive arts group… they were calmer… In the usual time, you may see that they always scold others. It’s hard for them to control their emotions, but in the group, it’s obvious that they became more peaceful. |
|  | Happier  (n=1) | I think the group gave them lots of happy stuffs which helped substitute their unhappiness… so that they became happier and less upset, thereby in general having a better emotional well-being. |
|  | Less irritated  (n=1) | After he joined the group, I realized that he was obviously… less irritated… In the past, he would hit things when he felt unhappy…even though his hands got swollen and things were all broken…Such scenario no longer happened. |
|  | Less mood swing  (n=2) | He was quite emotional… would easily lose his temper and hit himself… but in those ten weeks (the time the intervention program was held), I realized that he was less… emotional. |
| Expressivity  (N=8) | More verbal expression  (n=4) | (He became) More expressive, easier to make him talk… In the past, when you asked him something… if he didn’t want to think or answer, you need to prompt him a lot in order to make him talk. But now, you don’t need to push so much. |
|  | Express through arts  (n=4) | What impressed me the most is… there was a participant with autism. He rarely speaks. Once he drew… he drew his father. But his father had never come to our centre. The one who always comes to pick him up is his mother. When asking him to draw someone that is important in his life, he drew his father. At that moment… I realized that actually he has many things in his mind. Through the expressive arts intervention group, I can know more about the participant. |
| Self-regulation  (N=10) | Improved emotional regulation  (n=6) | When I feel sad, I told and share it with others… It makes me feel happier.  When I am upset, I would listen to music. |
|  | Improved in behaviors  (n=4) | There is a male participant who is very impulsive. He would easily lose his temper and hit others when he felt unsatisfied… But I realized that… when he joined the group, his ability to control his emotion and behaviors got improved. |
| Interpersonal relationship  (N=8) | Improved relationship with others  (n=5) | There is more connection between the six participants who had participated in the group. For example, they would remind each other when it’s time for joining the group… Interaction and communication increased among them. |
|  | Improved social skills  (n=3) | I realized that two of the participants learnt to “wait”. They gradually understand that… when other is talking, they need to wait until he/she finishes… This is respect… In the past, they just ignored you and kept saying what they wanted to say… Ignoring how you felt. |

*Note*. N = number of all coding in each cluster of themes; n = number of all coding in each group of codes.
